# Integrating Host Transcriptomics and Metagenomics in the Periparturient Dairy Cow: From Molecular Signatures to Host–Microbiome Interfaces

**DOI:** 10.3390/vetsci13101020

**Published:** 2026-09-26

**Authors:** Qingshan Ma, Mohamed Tharwat, Abd Ullah, Junjie Liu, Abdulrahman A. Alkheraif, Muhammad Zahoor Khan

**Affiliations:** 1 College of Agriculture and Biology, Liaocheng University, Liaocheng 252000, China; 2 Department of Clinical Sciences, College of Veterinary Medicine, Qassim University, P.O. Box 6622, Buraidah 51452, Saudi Arabia; atieh@qu.edu.sa; 3 Department of Pathology and Laboratory Diagnosis, College of Veterinary Medicine, Qassim University, P.O. Box 6622, Buraidah 51452, Saudi Arabia

**Keywords:** transition cow, transcriptomics, rumen and hindgut microbiome, negative energy balance, metagenome, immunometabolism, multi-omics integration, ketosis, metritis

## Abstract

The periparturient or transition period—roughly three weeks before to three weeks after calving—compresses the most severe metabolic, endocrine and immunological demands of the production cycle into a few weeks and accounts for most clinical disease and involuntary culling in dairy herds. Conventional surveillance relies on downstream indicators—non-esterified fatty acids, β-hydroxybutyrate, calcium and acute-phase proteins—that document failed adaptation without resolving its regulatory origins. Two omics platforms now close that gap. Transcriptomics shows that the liver, adipose tissue, mammary gland, endometrium, rumen epithelium and circulating leukocytes each execute a distinct, temporally ordered program organized around lipid mobilization, gluconeogenesis, endoplasmic reticulum stress, redox defense and inflammatory resolution, layered over a regulatory tier of non-coding RNAs. Metagenomics shows in parallel that rumen, hindgut, uterine, vaginal, teat-apex and colostrum communities are restructured by parturition and by the dietary shift into lactation, with reproducible dysbiotic signatures accompanying ketosis, fatty liver, metritis, endometritis and intramammary infection. Critically, the two layers are mechanistically coupled: microbial short-chain fatty acids, secondary bile acids, purine derivatives and amino acids reprogram hepatic and immune cell transcription, while host lipolysis, inflammation and mineral demand reshape community structure. This review synthesizes both groups of literature within a single framework, formalizes five host–microbiome interfaces supported by paired measurements and identifies the biomarker, nutritional and genetic-selection opportunities that emerge only when host and microbial evidence are read together.

## Simple Summary

The three weeks before and after calving are the most demanding phase of the dairy cow’s production cycle, and most cases of ketosis, fatty liver, uterine infection and mastitis arise within this window. Herd monitoring still depends on blood indicators that confirm impaired adaptation only after it has occurred. Two complementary technologies now allow the underlying regulation to be examined directly: transcriptomics, which measures gene activity in tissues such as liver, adipose tissue, mammary gland and uterus, and metagenomics, which characterizes the microbial communities of the digestive tract, reproductive tract and udder. This review evaluates the two fields together and shows that they are mechanistically linked, since microbial products influence tissue gene activity, while the metabolic and inflammatory state of the cow reshapes microbial composition. Considered jointly, the evidence indicates earlier diagnostic markers and nutritional and genetic strategies directed at preventing transition-period disease rather than detecting it.

## 1. Introduction

The periparturient period spans approximately three weeks before to three weeks after parturition and is the most physiologically demanding phase of the dairy cow’s production cycle [1,2,3]. The cow must complete fetal growth, initiate copious milk synthesis and re-establish uterine and metabolic homeostasis while dry matter intake fails to keep pace with nutrient and energy requirements [4]. Most metabolic disorders—milk fever, ketosis, retained placenta and displaced abomasum—and infectious diseases such as mastitis and metritis appear within two weeks of calving [5,6], and because transition performance predicts subsequent yield, fertility and longevity, the period is regarded as the single most important determinant of lifetime productivity and herd profitability [2,7].

As intake declines, cows enter negative energy balance (NEB), estimated to affect 30–50% of the milking herd and sustained by extensive mobilization of body reserves [1,8], so adipose triacylglycerol is mobilized, and hepatic gluconeogenesis and ketogenesis increase [9,10,11]. Non-esterified fatty acids (NEFAs) and β-hydroxybutyrate (BHB) are the principal metabolites of this mobilization and adversely affect multiple bovine cell types [12]; where hepatic capacity for complete fatty acid oxidation is exceeded, ketone bodies accumulate, and triacylglycerol is deposited in hepatocytes [13,14,15]. This load is compounded by falling calcium, sharp shifts in estrogen, progesterone and cortisol and suppression of neutrophil and lymphocyte function, termed periparturient immunosuppression [16,17,18,19]; consistently, plasma amino acid and tryptophan metabolism are altered across the transition and correlate with immune markers [20].

NEB and hypocalcemia also promote a pro-oxidant state now considered a central mechanistic link between metabolic dysfunction and impaired inflammatory responses [21,22]. Intervention work reinforces this: selenium and vitamin E status modulates periparturient antioxidant capacity and health outcomes [23], and combined selenium, vitamin E and folate supplementation attenuates bovine mastitis [24]. Yet monitoring still relies on NEFAs, BHB, calcium, haptoglobin, body condition score and clinical signs of uterine disease [8,25], downstream endpoints that show adaptation has failed without identifying the regulatory events, at the level of gene expression or of the resident microbial community, that drive that failure.

This is the principal rationale for transcriptomics. Genome-wide hepatic profiling shows that thousands of genes are differentially expressed within days of calving, revealing coordinated shifts in fatty acid oxidation, cholesterol metabolism and inflammatory signaling that conventional biomarkers cannot resolve [26,27,28,29,30], with comparable reprogramming in adipose tissue [31] and in the endometrium and leukocytes, where altered expression is linked directly to subclinical endometritis and impaired neutrophil function [19,32].

A parallel and, until recently, less appreciated dimension occurs in the microbial ecosystem. The shift from a forage-based far-off ration to an energy-dense lactation diet restructures ruminal bacterial and archaeal composition within days [33], superimposed on marked inter-animal variation present even under stable conditions [34]. These shifts are functionally consequential: rumen microbial genes and metabolites associate with early-lactation performance through a ruminal-to-systemic metabolite axis [35], and the gut microbiome shapes circulating bile acid pools regulating immune cell function during excessive lipolysis [36]. Beyond the digestive tract, healthy uterine involution and metritis are distinguished by characteristic community signatures [25,37].

Critically, the two systems are coupled: microbial metabolites shape immune gene expression, and host metabolic status shapes community structure [35,36], so a systems-level understanding requires joint consideration rather than separate fields of enquiry [38]. Yet the two groups of literature have developed largely in parallel. This review addresses that gap, summarizing tissue-resolved transcriptomic responses, the metagenomic reconfiguration of the major host-associated niches and the nutritional and management levers acting on both, before formalizing the host–microbiome interface and its translational consequences. The architecture of the period is summarized in Figure 1.

## 2. Literature Search Strategy

This article is a narrative review. No systematic search protocol was applied, and no review protocol was registered; neither PRISMA-based screening nor formal risk-of-bias assessment was undertaken. The literature was assembled through structured but non-exhaustive searching, and the synthesis is therefore interpretive rather than quantitative.

PubMed/MEDLINE, Web of Science and Scopus were searched up to August 2026, with Google Scholar and ResearchGate used additionally to retrieve full texts and to identify sources not returned by the three indexed databases. No date restriction was applied at the search stage; prioritization by publication year took place during selection (below). Searches combined population terms (dairy cow, dairy cattle, periparturient, peripartum, transition period and early lactation) with platform terms (transcriptome, RNA-seq, differential gene expression, microRNA, circRNA, metagenome, metagenomics, 16S rRNA, microbiota, microbiome and multi-omics) and context terms (liver, adipose tissue, mammary gland, endometrium, leukocyte, rumen epithelium, hindgut, uterus, colostrum, negative energy balance, ketosis, fatty liver, metritis and mastitis). Reference lists of the retrieved articles and of prior reviews were screened manually for further sources.

Only articles published in English in peer-reviewed journals indexed in the Science Citation Index Expanded were considered. Reports were retained when they sampled dairy cows within a defined window relative to calving, reported host transcriptomic or host-associated microbial community data generated by sequencing and stated the comparison or model and the molecular outcome explicitly. Conference abstracts, preprints and reports without full data were excluded. Work in rodents, in other ruminant species and in vitro was cited only where it established a mechanistic link with no bovine equivalent, and the model system is identified explicitly at each such point.

The review is anchored on work published between 2020 and 2026, the period over which sequencing-based transcriptomic and metagenomic profiling became routine in cattle; this window supplies 93 of the 139 sources cited (67%), and the primary microbial community studies synthesized here were published almost exclusively from 2022 onwards. The earlier literature was retained deliberately rather than removed by a date filter. The remaining 46 sources, published between 1989 and 2019, are cited where they are required to support the discussion: the foundational descriptions of transition physiology, negative energy balance, hepatic lipid metabolism and periparturient immunosuppression against which the recent omics findings are interpreted, as well as earlier tissue-level transcriptomic work that predates the current sequencing era. The search and selection process is summarized in Table 1.

## 3. Transition Period as a Host–Microbiome Systems Problem

The periparturient period is best framed as a systems problem in which the host and its resident microbial communities respond in concert to a single, compressed metabolic and endocrine transition. On the host side, the coordinated shift from storage to output is executed across the liver, adipose tissue, mammary gland, endometrium, rumen epithelium and circulating leukocytes; on the microbial side, parturition and the ration change simultaneously restructure the rumen, hindgut, uterine, vaginal and udder-surface communities. These two arms are not independent: microbial fermentation products (short-chain fatty acids, secondary bile acids, amino acids and purine derivatives) reprogram host transcription in liver and immune cells, while host lipolysis, acute-phase inflammation and mineral demand reshape microbial community structure and function. This review therefore treats transcriptomic and metagenomic evidence as two windows on one coupled system and organizes the synthesis around the pathway nutrition → ruminal fermentation → microbiome → metabolites → hepatic metabolism → immune response → performance.

## 4. Host Transcriptomic Signatures of Periparturient Adaptation

Transcriptomics has been the workhorse platform for the mechanistic study of transition biology because the tissues of interest have well-characterized roles and can be sampled by biopsy [39,40]. The studies detailed in Table 2 are consistent in one respect: the transition transcriptome is not a diffuse stress response but a structured reallocation of transcriptional capacity from storage and growth toward oxidative energy production, coupled to a redox and inflammatory program whose resolution—not its initiation—separates healthy from diseased animals.

### 4.1. Liver: The Metabolic Command Center

The liver executes the most legible program. Sequencing at day −21 versus day +7 identified 198 differentially expressed genes (DEGs), upregulating lipid handling, gluconeogenesis and cell division while repressing glycogen, polysaccharide and ketone metabolism, an unambiguous prioritization of energy output over storage [41]. Deeper sampling at day −22 and days +10 and +17 yielded 1063 DEGs enriched for lipid metabolism, cholesterol homeostasis and gluconeogenesis, 138 of them novel for transition cows, with hubs linking cholesterol and fatty acid pathways under AMPK control and suggesting that hepatic lipoprotein export is rate-limiting [42].

Superimposed on this is a proteostatic program: thirteen out of fourteen unfolded protein response genes rose 1.9- to 4-fold at one week postpartum before declining, with spliced *XBP1* confirming IRE1 activation [43]. Endoplasmic reticulum (ER) stress is thus a defining but self-limiting feature of the first postpartum week, and its persistence plausibly links elevated NEFA and inflammation to fatty liver. Cows with high hepatic *FGF21* upregulated ER stress, inflammation and NFE2L2-dependent cytoprotection despite indistinguishable intake, energy balance and yield, implying that FGF21 primes the liver for stress adaptation rather than driving metabolic flux [44].

Prepartum energy supply programs this capacity: restriction enhanced oxidative phosphorylation, amino acid catabolism and fatty acid oxidation with greater activation of the lipid-oxidative and antioxidant regulators listed in Table 2, whereas overfeeding favored triglyceride synthesis and higher postpartum liver triacylglycerol [45]. Overfed cows that later developed clinical ketosis differed from those that did not by 3065 DEGs, with impaired gluconeogenesis and inhibited mTOR and insulin signaling [46]: susceptibility is transcriptionally visible before disease. Clustering by susceptibility to lipid-related metabolic disorders implicated glutathione metabolism, eicosanoid pathways and differential MHC class II and interferon-inducible genes, shifting the framing toward immunometabolism and hepatic immune surveillance [47].

Transcriptomic phenotyping also discriminates among cows at the same physiological stage: over-conditioned animals stratified by serum metabotype showed DEGs separating cows whose insulin, IGF-1, leptin, adiponectin, haptoglobin and glucose values were statistically indistinguishable, and among three metabolic clusters at day 21, those with a normal-like metabotype preserved their liver function despite the excess condition [48]. Hepatic transcription also predicts productivity, with the intersection of 795 DEGs with co-expression modules yielding eight hub genes that explained milk yield with R^2^ = 0.946 [49], while multiparous cows upregulated gluconeogenesis but downregulated immune genes, antimicrobial peptides and 22 nutrient transporters alongside senescence markers, showing accelerated hepatic aging that plausibly explains the greater disease susceptibility and shorter longevity of older cows [50].

Several regulators and contexts modulate this program. In precision-cut liver slices, PPARδ and PPARγ agonists upregulated fatty acid degradation and ketogenesis, while PPARγ uniquely inhibited cytokine, TLR and antigen presentation pathways [51]. The body condition conditions the response to the feeding level, with thin grazing cows requiring overfeeding to activate protective glutathione and taurine pathways, whereas optimally conditioned cows benefited from restriction [52]. Fertility-divergent cows differed in 807 hepatic and 815 muscle DEGs, with low-fertility animals failing to resolve inflammation postpartum [53]. Hepatic responsiveness itself depends on rumen health, with significant DEGs appearing only in cows without subacute ruminal acidosis [54]: the first clear signal that the microbial layer gates the host program. At finer resolution, single-nucleus analysis of type II ketosis resolved 14 hepatic cell types and identified central venous hepatocytes as pivotal, with a ketone body–butyrate–*HADHA* axis as the core pathogenic mechanism, since *HADHA* silencing rescued NEFA-treated hepatocytes [55]. Underlying much of this, insulin resistance signaling is consistently upregulated under NEB [56,57,58].

Nutritional inputs are read directly by the hepatic transcriptome. Rumen-protected choline and methionine produced 1123 DEGs, choline favoring fatty acid synthesis and glucose and lipid pathways, while methionine engaged the TCA cycle and inflammatory signaling [59]; comparing niacin with methionine, 1210 DEGs showed niacin acting directly on fatty acid synthesis and transport genes, while methionine acted indirectly as an NAD/NADP precursor [60]. These divergent mechanisms rationalize individualized supplement selection and align with evidence that rumen-protected limiting amino acids and choline improve immune, antioxidative and inflammatory status [61] and that methionine and choline together enhance metabolism and milk production performance [62]. Dose matters: high-dose niacin upregulated acute-phase and MHC class II transcripts while downregulating 338 metabolic transcripts and 19 miRNAs, potentially amplifying an already pro-inflammatory state [63], whereas prepartum phosphorus restriction produced only 175 and 165 DEGs and no phenotypic impairment, indicating it can be applied safely to mitigate hypocalcemia [64].

### 4.2. Adipose Tissue: An Endocrine and Immune Organ

Adipose tissue has been reconceptualized from a passive depot to an active endocrine and immune organ, largely based on omics evidence [40]. Mobilization releases adipokines and cytokines and promotes immune infiltration and adipocyte death [65]; *LIPE* is elevated in ketosis and fatty liver, and *DGAT1* regulates lipolysis and autophagy in ketotic cows [66], with enhanced autophagy accompanying increased lipolysis in fatty liver [67]. Oxylipids and endocannabinoids couple the adipose state to immune competence [68,69], reframing transition disorders as immunometabolic rather than purely energetic.

Temporal profiling identified 1946 and 1524 postpartum DEGs spanning lipid metabolism, inflammation and redox signaling, resolving lipolysis triggers, lipid synthesis inhibitors and mediators bridging inflammation to reactive oxygen species [31], while dry-period overfeeding produced 2434 DEGs prepartum that waned after calving, with PPARγ as a key upstream regulator [70]. Disease signatures are well defined: downregulated glyceroneogenic enzymes indicate impaired re-esterification, with prepartum *CD209* (a candidate predictive biomarker) and postpartum tenascin C implicating adipose fibrosis [71], while *NTRK2*, encoding the BDNF receptor TrkB, emerged as a downregulated hub gene, implicating neurotrophic control of excessive lipolysis [72]. Over-conditioning also acts on non-coding, differentially expressed miRNAs, shifting metabolism toward catabolism and impaired insulin sensitivity [73]. The tissue is directly manipulable, with oleic acid infusion limiting lipolysis and improving insulin sensitivity through PLIN5 and PPARα [74]. Finally, severe NEB produced 1209 adipose DEGs, and the chemokine *CCL21*—reduced in tissue and plasma, with its receptor expressed in granulosa cells and recombinant protein suppressing progesterone secretion in vitro—emerged as a biomarker for NEB-associated subfertility [75].

### 4.3. Mammary Gland

Circular RNA profiling across perinatal and dry periods showed predominant upregulation, enrichment of phosphatidylinositol 3-kinase binding and enhanced immune pathways including cell adhesion molecules, with competing endogenous RNA analysis indicating miRNA sponging [76]. Redox defense is coordinated here, with phosphorylated NFE2L2 rising postpartum without changes in the total protein alongside upregulated glutathione and thioredoxin system genes and their transcriptional regulators [77]. Endocrine and dietary manipulation reveals plasticity: prolactin suppression produced 866 DEGs, upregulating AMPK signaling while downregulating lipid metabolism and reducing mammary development [78], whereas linseed upregulated cell cycle and immune system development and downregulated lipid and carbohydrate metabolism, with hepatic *GLUT2* rising postpartum, consistent with greater glucose release for lactose synthesis [79]. The gland is also an entry point for systemic disturbance: after an intramammary lipopolysaccharide challenge, mammary tissue activated NOD-like, Toll-like and RIG-I-like receptor pathways, while the liver inhibited mitochondrial fatty acid elongation and accumulated triacylglycerol [80]: mastitis drives hepatic lipid accumulation rather than remaining local. Circadian cues matter too, with chronic light–dark phase shifts in late gestation upregulating hepatic cholesterol biosynthesis and ER stress markers while reducing mammary extracellular matrix genes, lipid transporters and oxidative phosphorylation [81].

### 4.4. Endometrium and the Reproductive Tract

Metritis and its associated uterine microbiome are accompanied by endometrial DEGs persisting into mid-lactation, long after the microbiome has normalized [82], a molecular explanation for the carryover effect on fertility. Profiling at 7 and 21 days postpartum showed 73 genes and 31 miRNAs distinguishing cows developing cytological endometritis; healthy cows transitioned robustly from pro-inflammatory to tissue repair pathways, whereas affected cows showed only 31 DEGs across time, indicating arrested resolution alongside distinct uterine communities and elevated acute-phase proteins [83]. The pathological signal is failure to resolve, not failure to initiate, inflammation. Parity modulates this, with metritic cows downregulating *IGF1*, while healthy multiparous cows expressed more *PTX3*, *TNFα* and *SERPING1* than primiparous animals [84]. Nutritional status acts with cell-type specificity: laser-capture microdissection showed 1049 stromal, 24 glandular and 52 luminal DEGs under severe versus mild NEB, with stromal downregulation of interferon-stimulated genes critical for maternal recognition, glandular chemokine overexpression and luminal under-expression of adaptive immunity genes [85]. The trace mineral form modifies both compartments, amino acid-complexed sources enhancing leukocyte bactericidal capacity and endometrial TLR/NF-κB, prostaglandin, antioxidant and mucin expression [86], aligning with evidence that vitamins, rumen-protected amino acids and trace minerals act coordinately to prevent periparturient mastitis [87]. Beyond the uterus, mild undernutrition produced 293 oviductal DEGs but almost none in the endometrium or corpus luteum [88], and severe NEB downregulated granulosa cell genes for cellular organization, proliferation and fatty acid metabolism [89], while circulating miRNAs driving PI3K-Akt, NF-κB and JAK-STAT signaling discriminate metritis and impair angiogenesis and repair [90].

### 4.5. Circulating Leukocytes and Innate Immune Cells

Leukocyte transcriptomics is attractive because blood is minimally invasive and repeatedly sampleable, making longitudinal and, ultimately, on-farm application feasible [39]. Among 170 cows at 14 days in milk, low circulating IGF-1 predicted lower intake and energy balance and more health disorders, with 1032 DEGs revealing a switch from oxidative phosphorylation to aerobic glycolysis and upregulated antimicrobial peptides, whereas high-IGF-1 cows expressed more T- and B-cell genes, a trade-off between energy-intensive innate defense and adaptive competence [39]. Neutrophil profiling sharpens this, with 3500 DEGs in high-metabolic-risk cows showing downregulated recruitment, interferon signaling and apoptosis with upregulated survival genes [91]. Capacity is time-dependent: cows vaccinated before calving showed 2235 whole-blood DEGs versus and only 208 after calving, with the postpartum group uniquely upregulating *ARG1*, consistent with M2-like immunosuppression [92], so periparturient immunosuppression is partly a failure of metabolic flexibility and arginine availability. It is nonetheless addressable, with pegbovigrastim upregulating recognition, adhesion, antimicrobial and inflammasome genes while downregulating *IL8* and *TNF* [93]. Breed background independently structures the program, with Holsteins upregulating pathogen recognition genes prepartum, while Brown Swiss showed higher postpartum migration and antimicrobial and oxidative stress gene expression [94]. One-carbon nutrition acts on the same axis, with coated folic acid upregulating TNF, NF-κB and antigen processing pathways, with the low 120 mg dose giving more balanced immune activation than the high dose, [95] and increasing milk yield while enriching glycolysis, folate biosynthesis and glutathione metabolism [96]; this is consistent with evidence that folate, selenium and vitamin E jointly support periparturient udder health [24]. In six periparturient cows sampled from −20 to +7 days, leukocytes shifted from prepartum lipid metabolism and phagosome programs to postpartum immune and endocrine activation (281 DEGs) with concurrent suppression of lipid and amino acid metabolism; plasma BHBA was associated inversely with DNA replication and cell-cycle pathways, while IL-1β tracked with cellular energy metabolism, positioning metabolite load as a proximate driver of leukocyte transcriptional capacity [97].

### 4.6. Rumen Epithelium, Muscle and Corium

Rumen papillae biopsied at −3, +1 and +6 weeks showed 1011 and 729 DEGs despite increased epithelial sloughing, with desmosome assembly, EGF, TGF-β and IGF-axis pathways as central hubs, a blueprint of epithelial turnover across the dry-to-lactation shift [98]. In skeletal muscle of ketotic cows, two circular RNAs sponge five miRNAs regulating targets within the MAPK, Wnt, FoxO, TNF, mTOR and autophagy pathways, enhancing mitochondrial ATP production and reducing ketone accumulation [99]. In the corium, amino acid-complexed trace minerals reduced heel horn erosion, improved biotin availability and enhanced antioxidant defense [100]. In seven multiparous Holsteins followed through the transition, ruminal epithelium upregulated ketogenic and monocarboxylate transport genes while downregulating innate-immune receptor and tight-junction genes; concurrently; concurrently, *Anaerovibrio lipolytica*, *Prevotella bryantii* and *Megasphaera elsdenii* expanded at the expense of *Fibrobacter succinogenes* and *Butyrivibrio proteoclasticus*, raising butyrate and lowering pH and acetate, linking diet-driven microbial shifts to epithelial adaptation under negative energy balance [80].

### 4.7. The Non-Coding Layer and Cross-Tissue Integration

A consistent tier of microRNAs and circular RNAs modulates the coding response. Hepatic miRNA sequencing identified 53 expressed miRNAs dominated by miR-122 and miR-192, with miR-143 downregulated under severe NEB and its lipid-handling target *LRP2* upregulated [101]; array profiling validated five upregulated miRNAs associated with non-alcoholic fatty liver disease, with putative targets in fatty acid desaturation and innate immune signaling [102]. With adipose responses to over-conditioning [73], mammary sponge networks [76], the muscle circRNA axis [99] and circulating metritis signatures [90], these establish post-transcriptional control as an integral, diagnostically accessible and nutritionally responsive component of transition biology [63]. Meta-analysis across adipose tissue, blood, the endometrium, the hypothalamus and the liver further revealed tissue-specific innate immune responses to NEB; the endometrium uniquely downregulated defense gene sets, while other tissues upregulated them, with *LBP*, *PTX3*, *S100A12* and *LCN2* bridging metabolism and immunity [103], precisely the configuration permitting opportunistic uterine colonization.

**Table 2:** Tissue-resolved transcriptomic signatures of the periparturient dairy cow.

Tissue	Comparison/Model	Sampling Window	Principal Molecular Findings	Interpretation	Reference
Adipose	Transition time course	−11, +6, +13 d	1946/1524 DEGs; *TLR4*, *IL6*, *FASN*, *ELOVL6*, *C3*, *CXCL2*, *HMOX1*	Lipolysis, inflammation and ROS are one network	[31]
Leukocytes	High vs. low IGF-1	14 DIM	1032 DEGs; *PKM2*, *PDK4*; *S100A8/A9*, *DEFB*; *BOLA-DQB*, *MS4A1*	Innate–adaptive trade-off indexed by IGF-1	[39]
Liver	Transition, multiparous Holstein	−21 vs. +7 d	198 DEGs; lipid handling, gluconeogenesis, cell division up; glycogen/ketone metabolism down; p53 down	Metabolic prioritization of energy output over storage	[41]
Liver	Transition, Red Holstein	−22, +10, +17 d	1063 DEGs; hubs *CYP7A1*, *APOA1*, *HMGCR*, *FDFT1*, *SQLE*; AMPK enrichment; *GPX3*, *G0S2*	Cholesterol–fatty acid coupling and AMPK-governed adaptation	[42]
Liver	Time course, UPR panel	−3, +1, +5, +14 wk	13/14 UPR genes up 1.9–4×; spliced *XBP1*; *ATM*, *MSH2*, *XRCC5*	Self-limiting ER stress peaking at wk 1 postpartum	[43]
Liver	High vs. low *FGF21*	wk 1 postpartum	ER stress, inflammation, NFE2L2 cytoprotection up; metabolome largely unchanged	FGF21 primes stress adaptation, not metabolic flux	[44]
Liver	Overfed vs. energy-restricted	Prepartum → postpartum	RE: oxidative phosphorylation, FA oxidation, *PPARA*, *SREBF1/2*, *NFE2L2*; OF: TAG synthesis	Prepartum energy programs hepatic oxidative capacity	[45]
Liver	Overfed → clinical ketosis vs. not	Prepartum	3065 DEGs; *FBP2*, *PDK4* down; *SREBF2*, bile acid synthesis up; mTOR/insulin inhibited	Disease susceptibility is transcriptionally pre-visible	[46]
Liver	Susceptible vs. resistant to LRMD	+1 d and later	*GSTA1/A4/T1*; eicosanoid *PLA2*, *CYP2J2*; *BOLA-DQB*, *IFI6/27/44*	Immunometabolic rather than purely lipid basis of susceptibility	[47]
Liver	Metabotype stratification	Postpartum (+21 d)	DEGs separate cows with identical endocrine panels 48 and 24 DEGs; *APOA1*, *FGF21* down; *MAOA*, *CAV2*, *RASSF2* up; *SLC12A8*, *SLC16A5* down	Transcriptome resolves beyond standard biomarkers Over-conditioned cows can retain adaptive metabotypes	[48]
Liver	High vs. low milk yield	+21 d	795 DEGs; hubs *CXCL17*, *HES6*, *SHISA2*, *ERN2*, *IL1RL1*, *PMP22*, *FBXL22*, *CYP26C1*; R^2^ = 0.946	Hepatic signature predicts lactation potential	[49]
Liver	Primiparous vs. multiparous	~+14 d	568/719 DEGs; *PC*, *FBP2* up; *LYZ2/3*, *NLRP6*, 22 *SLC* down; *CDKN2A*, *HSPA1A* up	Accelerated hepatic aging with parity	[50]
Liver	PPAR agonists, precision-cut slices	−8, +7 d	*ACADVL*, *PDK4*, *PLIN2*, *ACSL1* up; PPARγ inhibits *TLR4*, *IL21R*; 91 novel PPAR targets	PPARγ couple’s lipid handling to immune restraint	[51]
Liver	BCS × feeding level, grazing	Prepartum	BCS4 3× more DEGs; *BCAT1*, *GPX3*, *ALB*, *FABP7*	BCS-conditional feeding strategy	[52]
Liver + muscle	Fertility-divergent genotypes	Transition	807/815 DEGs; *S100A8/A9*, *SAA3*, *HP*; *IGF1*, *PDK4*, *CPT1B*	Unresolved inflammation underlies subfertility	[53]
Liver	SARA vs. non-SARA	Postpartum	DEGs only in non-SARA; *PC*, *G6PC*, *PCK1*, *APOA1* up; *INSR*, *PPARG* activated	Rumen health gates hepatic transcriptional response	[54]
Liver	snRNA-seq, type II ketosis	Postpartum	14 cell types; central venous hepatocyte loss; ketone–butyrate–*HADHA* axis	Cell-type-resolved pathogenic node	[55]
Adipose	Dry-period overfeeding	−14, +1, +14 d	2434 DEGs prepartum; PPARγ, mTOR, *CXCR4*, *IGF1*	Prepartum intake programs adipose anabolism	[70]
Adipose	Ketotic vs. non-ketotic	−21, +10 d	*PCK1*, *PCK2* down; *CD209* prepartum; *TNC* postpartum	Impaired glyceroneogenesis and adipose fibrosis	[71]
Adipose	Type II ketosis, WGCNA	+10 d	*NTRK2*/TrkB hub gene downregulated	Neurotrophic control of adipose lipolysis	[72]
Adipose	Over- vs. moderate BCS, miRNA-seq	−49, +21 d	10/43 DE-miRNAs; miR-486; JAK-STAT, PI3K-AKT targets	Non-coding programming of adipose catabolism	[73]
Adipose	Oleic acid infusion	−12, +7, +14 d	PLIN5, PPARα up; *SDHA*, *SIRT3*, *SOD2*, *PARP3*; osteopontin down	Nutritional restraint of lipolysis	[74]
Adipose	Severe vs. moderate NEB	Transition	1209 DEGs; *SCD*, *BHLHE40*; *CCL21*/*CCR7* axis	Adipose signal for NEB-associated subfertility	[75]
Mammary	circRNA profiling	Perinatal, dry	PI3K binding, cell adhesion molecules; ceRNA sponging	Non-coding control of mammary immunity	[76]
Mammary	NFE2L2 signaling	−30, 1, 15, 30 d	p-NFE2L2 up; *GSR*, *GCLC*, *GPX1*, *TXN*, *TXNRD1*, *NQO1*	Coordinated antioxidant mobilization	[77]
Mammary	Prolactin suppression	L1, dry, L2	866 DEGs; AMPK up; *INSIG1*, *SREBF1*, *SCAP*, *STAT3*	PRL-mediated metabolic and immune adaptation	[78]
Mammary + liver	Intramammary LPS	2.5 h post-challenge	189/107 DEGs; NLR, TLR, RLR up; *ACAA2*, *HMGCS2* down	Mastitis drives hepatic lipid accumulation	[80]
Liver + mammary	Circadian phase shift	−21 d	*HMGCR*, *SQLE*, *HSP90B1* up; *CTSB*, *COL1A1*, *CD36* down	Circadian disruption raises fatty liver risk	[81]
Endometrium	Persistent post-metritis change	To mid-lactation	DEGs persist after microbiome normalization	Molecular basis of carryover infertility	[82]
Endometrium	Cytological endometritis	7, 21 DPP	*IL1A/B*, *IL6*, *IL17A*, miR-200; arrested resolution	Failure to resolve, not to initiate, inflammation	[83]
Endometrium	Metritis, parity	1, 3, 6 DPP	*MX2*, *MYH10* up; *IGF1* down; *PTX3*, *TNFα*, *SERPING1*	Impaired involution via IGF and prostaglandin signaling	[84]
Endometrium (LCM)	Severe vs. mild NEB	Day 15 cyclic	ST 1049, GE 24, LE 52 DEGs; *MX1*, *STAT1*, *ISG15* down	Cell-type-specific loss of receptivity	[85]
Oviduct/CL	Underfeeding	80 d postpartum	293 oviductal DEGs; *FABP5*, *CRABP2*	Oviduct is the nutritionally sensitive compartment	[88]
Granulosa cells	Severe vs. mild NEB	60 DIM	*KRT4*, *PPL*, *TACSTD2*, *VNN2* down	Impaired follicular competence	[89]
Circulating miRNA	Metritis	Postpartum	PI3K-Akt, NF-κB, JAK-STAT; *AKT3*, *VEGFA*, *HIF1A*	Minimally invasive metritis signature	[90]
Neutrophils	High vs. low metabolic risk	Postpartum	3500 DEGs; *CXCR2*, *SELL*, *STAT1* down; *BCL2* up	Metabolic stress impairs recruitment and resolution	[91]
Whole blood	Vaccination before vs. after calving	Peripartum	2235 vs. 208 DEGs; *PDK4*, *HIF1A* up; *ARG1* postpartum	Immunosuppression as metabolic inflexibility	[92]
Leukocytes	Pegbovigrastim	Peripartum	*CD14*, *TLR2/4*, *MYD88*, *NLRP3*, *MMP9* up; *IL8*, *TNF* down	Pharmacological restoration of neutrophil function	[93]
Leukocytes	Brown Swiss vs. Holstein	−21, +7, +28 d	Holstein *CD14*, *TLR4*; Brown Swiss *ITGAL*, *MMP9*, *ALOX15*	Breed-specific immune strategy	[94]
Rumen epithelium	Papillae adaptation	−3, +1, +6 wk	*DSG1*, *EGFR*, *TGFB1*, *GHR*, *IGFBP2*	Epithelial turnover blueprint	[98]
Muscle	Ketosis, circRNA	Periparturient	circ_30628, circ_CoQ2; *COQ2*, *ST3GAL6*, *PCCB*	Non-coding support of mitochondrial ATP output	[99]
Corium	Trace mineral form	Periparturient	*BTD* up; *KRT5*, *CYBB* down; *NFE2L2*, *SOD1*, *PPARA/D* up	Reduced hoof lesion risk	[100]
Liver	miRNA sequencing, NEB	Postpartum	miR-122, miR-192 dominant; miR-143 down, *LRP2* up	miRNA control of hepatic lipid metabolism	[101]
Liver	miRNA array, severe NEB	Postpartum	miR-17-5p, miR-31, miR-140, miR-1281, miR-2885 up; *FADS2*, *CD14*, HNF3γ targets	NAFLD-associated miRNA program	[102]
Multi-tissue	NEB meta-analysis	Transition	Hubs *LBP*, *PTX3*, *S100A12*, *LCN2*; endometrium uniquely suppressed	Tissue-divergent innate immune reprogramming	[103]

## 5. Microbial Adaptation: Metagenomic Signatures of the Periparturient Cow

Metagenomics captures a layer of periparturient biology—host-associated microbial structure and function—that host-centered measurements cannot reveal, spanning the rumen, hindgut, uterus, vagina, teat apex, udder skin and colostrum (Table 3).

### 5.1. Rumen

Parturition perturbs the rumen independently of diet: between 3 and 5 days before calving and the day of calving, *Lachnospiraceae, Acetitomaculum*, *Methanobrevibacter*, *Olsenella*, *Syntrophococcus*, *Lachnospira* and *Lactobacillus* increased, while *Prevotellaceae* decreased, with concurrent declines in acetic, propionic, butyric and caproic acids, implying that loss of *Prevotellaceae* contributes to postpartum NEB [104]. The community continues to reorganize thereafter: Christensenellaceae and *Methanobrevibacter* decline, while *Ruminococcaceae*, *Moryella*, *Pseudobutyrivibrio* and Prevotellaceae rise by day 7, and *Sharpea* and *Olsenella* increase by day 14, with short-chain fatty acids peaking at day 7 [105].

Disease signatures are informative but not uniform. In clinical ketosis, *Christensenellaceae, Ruminococcaceae, Lachnospiraceae* and *Prevotellaceae* were enriched and correlated with serum BHB, while *Succinivibrionaceae* decreased, with enriched butyrate and suppressed propionate production, a shift toward ketogenic and away from glucogenic precursor supply even under adequate intake [106]. By contrast, shotgun sequencing found no differences in diversity, composition or predicted function between hyperketonemic and control cows, although asparagine and p-cresol were greater in affected animals [107], arguing that ruminal function rather than composition carries the disease signal.

Metagenome-resolved work supplies causal candidates: in a ketosis model, rumen disturbance produced an alanine deficiency tracking the phenotype, binning identified *Ruminococcus_E bovis* as the responsible alanine-synthesizing taxon, and alanine supplementation alleviated ketogenesis in hepatocyte models by activating gluconeogenesis [108]. In long-lived, high-yielding cows, elevated rumen propionate and upregulated microbial amino acid, cofactor and vitamin pathways accompanied purine metabolites, mediating 65.56% and 67.55% of the positive effects of specific taxa on milk yield [109]. Host condition shapes the community in turn, with high prepartum body condition being associated with enriched Firmicutes genera and citrate cycle enrichment [110], while monitoring across 168 days showed that calving and an LPS challenge triggered substantial change: fiber-associated taxa buffering severity by maintaining energy supply and gut integrity (a microbial “airbag”) [111].

### 5.2. Hindgut and Fecal Communities

The hindgut is an active participant rather than a passive terminus. Prepartum fecal structure separated cows subsequently susceptible to subacute ruminal acidosis from unsusceptible animals despite identical fecal pH, dry matter and volatile fatty acids [112], and nutritional diarrhea proved likewise hindgut- rather than rumen-driven, with fecal composition differing with lower volatile fatty acids and higher ammonia nitrogen [113].

Bile acids provide the principal biochemical currency of this axis: 32 plasma bile acids were identified, with most rising postpartum and correlating positively with total antioxidant capacity and glutathione peroxidase and negatively with triglycerides, alongside declining fecal diversity and increased microbial choloylglycine hydrolase and 7-α-hydroxysteroid dehydrogenase [114]. In hyperketonemia, Verrucomicrobiota and *Proteobacteria* dynamics were linked to enhanced lipolysis, *Frisingicoccus* correlated with branched-chain amino acids and ketogenesis, and elevated taurodeoxycholic acid with upregulated secondary bile acid biosynthesis tracked blood BHB [115].

Oxidative status is microbially conditioned. Fecal microbiota transplantation established causality: community composition explained 43.1% of oxidative stress variation, high-stress cows showed reduced microbial metabolism of glutathione precursors, and recipient mice reproduced the donor phenotype [116]; independently, oral *Phascolarctobacterium succinatutens* raised the antioxidant capacity and activated Nrf2–Keap1 signaling in mice, with low-stress cows having elevated serum tryptophan metabolites and more short-chain fatty acid producers [117]. The hindgut also participates in mineral homeostasis and barrier failure: hypophosphatemia selectively remodeled hindgut but not rumen communities, with α-methyl-m-tyrosine identified as a candidate mediator of host phosphorus dynamics [118], while in fatty liver disease, ileal sequencing showed reduced diversity with the expansion of *Clostridium perfringens*, elevated virulence and resistance gene content and reduced tight junction expression, with gavage into high-fat-fed mice exacerbating steatosis [119].

Postpartum energy status is legible in the same compartment [120], whereas antimicrobial exposure is a comparatively weak driver, with intramammary ceftiofur producing only subtle functional change against a far stronger effect of the lactation stage [121]. Maternal status is transmitted: calves born to dams with subclinical ketosis showed slower growth, more scours and reduced gut microbial diversity by three weeks [122], and parallel work in dairy sheep with periparturient paresis identified acylcarnitines as pre-clinical biomarkers alongside enrichment of *Fusobacterium* and *Erysipelatoclostridium* [123].

### 5.3. The Reproductive Tract

Uterine metagenomics has converged on a reproducible dysbiotic signature. Shotgun sequencing across 24 commercial farms showed lower alpha diversity in metritis and purulent discharge samples, with seven of the twelve most abundant genera increased—including *Bacteroides*, *Fusobacterium*, *Porphyromonas* and *Prevotella*—and *Dietzia* and *Microbacterium* decreased [124]. Distinguishing clinical from subclinical disease, cytobrush metagenomics found no diversity differences yet greater abundance of *Fusobacterium*, *Peptoniphilus*, *Peptostreptococcus* and *Trueperella* in clinical endometritis, whereas subclinical disease showed no association with bacterial colonization [125]; amplicon work is consistent, with composition differing at three weeks postpartum, showing lower Firmicutes and *Bacteroidetes* and higher *Actinobacteria* in diseased cows [126].

Disturbance is not confined to disease. Endometrial metagenomics identified seven phyla, with *Firmicutes* and *Fusobacteriota* dominating with opposite oscillatory patterns suggestive of competition; high *Fusobacteriaceae* abundance occurred in clinically healthy cows, and 32 predicted pathways shifted, including reduced pyridoxal 5′-phosphate and teichoic acid biosynthesis at day 3 [127], a caution against purely taxonomic diagnosis. The vagina supplies both baseline and predictive information: healthy cows carried 54 species dominated by *Bacillus*, *Streptococcus* and *Staphylococcus* at mean mucosal pH 7.2 [128], whereas cows developing incomplete uterine involution showed lower early diversity with sustained enrichment of *Fusobacterium*, *Porphyromonas* and *Prevotella* through days 5–20 [129].

Causal ordering is emerging. Bayesian network analysis traced a directional path from prepartum body weight and weight loss through plasma fatty acids, leukocyte death and immune activation to postpartum polymorphonuclear extravasation, uterine metabolites of tissue damage and enrichment of *Fusobacterium*, *Porphyromonas* and *Bacteroides* [130]. Parity conditions susceptibility: in primiparous cows, a challenge model validated in multiparous animals did not significantly increase metritis incidence, although high-dose cows carried a 2.7-fold greater hazard [131]. Dietary modulation is feasible, with grape pomace reducing *Fusobacterium necrophorum* and *Trueperella pyogenes* and stabilizing the mycobiome with lower *Candida* prevalence [132].

### 5.4. Udder Surface, Colostrum and the Resistome

The udder surface carries predictive information: prepartum teat apex metagenomics showed that cases of postpartum *Staphylococcus aureus* intramammary infection had markedly higher odds of prepartum *S. aureus* DNA (OR = 38.9), while several commensals were protective and Microcin B17 genes were more abundant in other cases [133]. Udder skin communities are themselves destabilized by calving, with diversity falling at 1–2 weeks postpartum and more severely in cattle than yak, nominating this as a window for mastitis prevention [134], and across maternal niches colostrum, vaginal and fecal microbiomes were distinct, with energy metabolism biomarkers correlating negatively with *Pseudomonas*, *Christensenellaceae* and *Methanobrevibacter* [135]. Metagenomics also indexes public-health-relevant gene content: naringin reduced antibiotic resistance genes by up to 9.0% and virulence factors by up to 7.2%, including *mecA*, *tetM/tetO* and *rmtF*, by reducing Proteobacteria [136]; phytosterols increased microbial crude protein and propionate while enriching *Fibrobacter succinogenes* [137]; and at high altitudes, *Brassica rapa* L. increased milk yield and enriched cellulolytic taxa correlated with elevated short-chain fatty acids, while N-carbamoylglutamate increased Lachnospiraceae genera [138].

**Table 3:** Metagenomic and microbial community signatures across periparturient niches.

Niche	Method	Comparison	Key Taxonomic Findings	Functional/Metabolite Findings	References
Gut + blood monocytes	Metagenomics + scRNA-seq	Excessive lipolysis	*Bacteroides* sp. OF04-15BH, *Paraprevotella* spp., *Treponema* sp. JC4	Secondary bile acid synthesis; GPBAR1-linked monocyte suppression	[36]
Rumen	16S + GC-MS/MS	Pre- vs. day of calving	Lachnospiraceae, *Acetitomaculum*, *Methanobrevibacter*, *Lactobacillus* up; *Prevotellaceae* down	Acetate, propionate, butyrate and caproate all decline	[104]
Rumen	16S + targeted/untargeted MS	0, 7, 14, 21 d postpartum	*Christensenellaceae, Methanobrevibacter* down; *Ruminococcaceae, Sharpea*, *Olsenella* up	Propionate, butyrate and valerate peak at 7 d	[105]
Rumen	Metataxonomics + metagenomics	Clinical ketosis vs. non-ketotic	*Christensenellaceae, Ruminococcaceae, Lachnospiraceae* up; *Succinivibrionaceae* down	Butyrate pathways up; propionate pathways down	[106]
Rumen	Shotgun + metabolome	Hyperketonemic vs. not	No compositional difference	Asparagine and p-cresol up; citrulline and acetate down	[107]
Rumen + liver	Metagenomics, metabolomics, transcriptomics	Energy metabolism disorder	*Ruminococcus_E bovis* (MAG 189) enriched for amino acid biosynthesis	Alanine deficiency; supplementation restores gluconeogenesis	[108]
Rumen + rectum	Metagenomics + multi-fluid metabolomics	Long-lived high-yielding cows	*Acidaminococcaceae*, *Parabacteroides*	Guanosine and D-ribose-1-phosphate mediate 65–68% of milk yield effect; EPA axis	[109]
Rumen	16S + metabolomics	BCS 3.5 vs. 2.75 prepartum	*Firmicutes* genera enriched (*Anaerovibrio*, *Blautia*, *Schwartzia*)	Citrate cycle enrichment	[110]
Rumen + duodenum	16S + NMR, 168 d	Calving and LPS challenge	*Bifidobacterium*, *Butyrivibrio*, Ruminococcaceae up	Fiber fermenters buffer challenge (“microbial airbag”)	[111]
Feces	16S + qPCR	SARA-susceptible vs. unsusceptible	*Ruminococcus*, Prevotellaceae_UCG-001 down; *Streptococcaceae* up in susceptible	Prepartum community predicts postpartum SARA	[112]
Rumen + feces	16S	Nutritional diarrhea	Fecal *Frisingicoccus* only in non-diarrhoeic; rumen unchanged	Lower fecal VFA and higher ammonia N	[113]
Feces + plasma	16S + targeted metabolomics	Transition time course	*Alistipes*, *Ruminococcaceae_UCG_014* biomarkers	32 bile acids; choloylglycine hydrolase and 7α-HSDH up	[114]
Gut	16S, fecal + plasma metabolomics	Hyperketonemia	*Verrucomicrobiota, Proteobacteria* dynamics; *Frisingicoccus*	Taurodeoxycholic acid up; ketogenic pathways up	[115]
Gut	Multi-omics + FMT to mice	High vs. low oxidative stress	*Marasmitruncus*, *Ruminococcus* sp. CAG:724	Microbiota explains 43.1% of OS; reduced GSH precursor metabolism	[116]
Hindgut	16S + metagenomics + metabolomics	High vs. low oxidative stress	*Paludibacter propionicigenes*, *Phascolarctobacterium succinatutens*	Tryptophan metabolites; Nrf2–Keap1 activation in mice	[117]
Hindgut	Metagenomics + metabolomics	Hypophosphatemia	*Lachnospiraceae_NK3A20_group*, *Acetitomaculum*	α-methyl-m-tyrosine mediates P dynamics; glycerophospholipid shift	[118]
Ileum	Metagenomics + mouse transfer	Fatty liver disease	*Clostridium perfringens*, *Enterobacter cloacae*, *Vibrio alginolyticus*	Virulence and ARG enrichment; barrier failure; causal steatosis	[119]
Feces	16S	High vs. low postpartal BW change	*Monoglobus*, *Christensenellaceae_R-7* up; *Romboutsia*, *Turicibacter* down	Amino acid vs. carbohydrate pathway divergence	[120]
Feces	Shotgun + untargeted metabolomics	Intramammary ceftiofur; lactation stage	*Actinobacteria, Proteobacteria* higher in late lactation	Lactation stage outweighs antibiotic effect	[121]
Calf gut	16S	Maternal subclinical ketosis	*Butyricicoccus pullicaecorum*, *Gallibacterium anatis* up; *Sharpae azabuensis* down	Reduced diversity and slower growth in offspring	[122]
Feces (sheep)	16S + metabolomics	Periparturient paresis	*Fusobacterium*, *Erysipelatoclostridium* up; *Faecalibacterium* down	Acylcarnitines as pre-clinical biomarkers	[123]
Uterus	Shotgun, 24 farms	Metritis/purulent vs. control	*Bacteroides*, *Fusobacterium*, *Porphyromonas*, *Prevotella* up; *Dietzia*, *Microbacterium* down	Reduced alpha diversity in disease	[124]
Uterus	Shotgun	Clinical vs. subclinical endometritis	*Fusobacterium*, *Peptoniphilus*, *Peptostreptococcus*, *Trueperella*	Wnt/catenin pathway reduced; SCE not colonization-linked	[125]
Uterus/vagina	16S + culture	Endometritis vs. healthy	*Firmicutes, Bacteroidetes down; Actinobacteria* up at 3 wks	Culture and sequencing concordant	[126]
Endometrium	Metagenomics	Healthy transition cows	*Firmicutes* (*Clostridia*) and *Fusobacteriota* (*Fusobacteriia*) oscillate reciprocally	32 pathways shift; pyridoxal 5′-phosphate, teichoic acid down at d 3	[127]
Vagina	16S + culture	Healthy cows, 3 farms	54 species; *Bacillus*, *Streptococcus*, *Staphylococcus*; 87% Gram-positive	Mucosal pH 7.2; broad antibiotic sensitivity	[128]
Vagina	16S, serial	Incomplete uterine involution	*Fusobacterium*, *Porphyromonas*, *Prevotella* up d 5–20; Firmicutes down	Candidate early-detection targets	[129]
Uterus + host	Bayesian network	Prepartum BW → metritis	*Fusobacterium*, *Porphyromonas*, *Bacteroides*	Directional path from BW loss to PMN damage to overgrowth	[130]
Uterus	16S, challenge model	Primiparous intrauterine challenge	*Fusobacterium*, *Trueperella* in metritis; *Escherichia* unassociated	Superior primiparous resistance	[131]
Uterus (beef)	16S + ITS	Grape pomace supplementation	*F. necrophorum*, *T. pyogenes* reduced; *Candida* spp. lower	Ascomycota-dominated mycobiome stabilized	[132]
Teat apex	Shotgun	Prepartum, *S. aureus* IMI outcome	*S. aureus* OR = 38.9; protective *C. efficiens*, *K. polaris*, *D. alimentaria*	Microcin B17 genes 99.7% prevalent	[133]
Udder skin	16S	Yak vs. cattle, periparturient	*Staphylococcus*, *Moraxella*, *Acinetobacter* core; *Acinetobacter* dominant in cattle	1–2 wk postpartum window of instability	[134]
Colostrum/vagina/feces	16S	Metabolism and mammary health	*Proteobacteria* in colostrum; *Firmicutes* elsewhere	NEFA, BHB negatively associated with *Pseudomonas*, Christensenellaceae	[135]
Rumen	Metagenomics (resistome)	Naringin supplementation	*Proteobacteria* reduced	ARGs −9.0%, VFs −7.2%; *mecA*, *tetM/tetO*, *rmtF*	[136]
Rumen	16S + metatranscriptomics + metabolomics	Phytosterol supplementation	*Fibrobacter succinogenes* enriched	Glycolysis, GH13 up; propionate up	[137]
Rumen	16S + LC-MS/MS	*Brassica rapa* L./NCG, high altitude	*Fibrobacter*, *Treponema*, *Prevotella*; *Anaerovibrio*, *Coprococcus*	SCFA up; arginine biosynthesis up; arachidonic acid down	[138]

## 6. Host Transcriptome–Microbiome Crosstalk (Multi-Omics Integration)

The preceding sections describe two response systems the literature has largely treated separately. The evidence now available makes that separation untenable. At least five mechanistically distinct interfaces can be identified, each supported by paired host and microbial measurements (Figure 2). The figure summarizes the pathway diet → microbiome → metabolites → tissues → gene expression → phenotype and the return arm host → metabolic environment → microbiome, so that transcriptomic and metagenomic evidence can be read as one system rather than two.

First, microbial glucogenic and amino acid supply gates hepatic gluconeogenesis. The clearest demonstration closes the loop from taxon to metabolite to host pathway to phenotype: *Ruminococcus_E bovis* supplies alanine, its deficiency tracks disease, and supplementation restores hepatocyte gluconeogenesis [108]. The fermentation data supply the context, since the postpartum decline in *Prevotellaceae* and propionate [104,105] and the ketosis-associated shift toward butyrate pathways [106] reduce glucogenic precursor supply precisely when hepatic gluconeogenic transcription is maximally induced [41,42]. Where induction fails, as in subacute ruminal acidosis [54], the microbial layer is the plausible upstream cause, while long-lived, high-yielding cows illustrate the constructive version of the same axis [35,109].

Second, microbial secondary bile acids reprogram immune cell transcription. Integrated single-cell sequencing, metagenomics and metabolomics in cows with excessive lipolysis showed activated secondary bile acid biosynthesis attributable to named taxa, decreased plasma conjugated bile acids and suppressed monocyte migration, phagocytosis and cytokine responses, plausibly via reduced GPBAR1 expression on CD14^+^ monocytes [36]. This is the most complete host-cell-resolution demonstration of microbial control over periparturient immune competence, dovetailing with leukocyte evidence that innate function is compromised under metabolic stress [39,91] and that the bile acid pool is itself dynamic across transition and tracks blood BHB in hyperketonemia [114,115].

Third, microbial metabolism sets the host redox capacity, which gates the transcriptional stress response. Transplantation established that gut communities explain 43.1% of oxidative stress variation through glutathione precursor metabolism, and short-chain fatty acid producers activate Nrf2–Keap1 signaling [116,117]. Precisely this axis is mobilized on the host side: NFE2L2 phosphorylation drives glutathione and thioredoxin gene upregulation in the mammary gland [77], *GPX3* is among robust hepatic transition DEGs [42], glutathione S-transferase expression separates susceptible from resistant cows [47], and NFE2L2 is differentially activated under prepartum energy restriction [45]. Microbial precursor supply and host NFE2L2 signaling are two halves of one redox system, reinforced by the benefits of selenium and vitamin E for periparturient antioxidant status [23].

Fourth, barrier failure couples microbial translocation to hepatic and systemic inflammation. Ileal dysbiosis with *Clostridium perfringens* expansion accompanied barrier failure and causally reproduced steatosis on transfer [119], the microbial counterpart of the host observation that hepatic unfolded protein response and inflammatory transcription peak in the first postpartum week [43,44] and that inflammatory challenge suppresses hepatic fatty acid oxidation while raising liver triacylglycerol [80]. Notably, the cross-tissue hub genes identified by meta-analysis are lipopolysaccharide- and pathogen-responsive [103], exactly what a translocation model predicts, and fiber-fermenting taxa appear to buffer the process [111].

Fifth, at the reproductive tract, causality runs from host tissue damage to microbial overgrowth and back to persistent transcriptional dysregulation. Bayesian analysis ordered these events explicitly [130], and the host precondition is transcriptionally visible, since the endometrium uniquely downregulates defense gene sets under NEB [103], and severe NEB suppresses interferon-stimulated genes in the stroma while driving glandular chemokine expression [85]. Once established, the community leaves a durable transcriptional scar, with endometrial DEGs persisting after microbiome normalization [82] and arrested inflammatory resolution accompanying distinct uterine communities [83], with parity-dependent immune competence conditioning whether challenge becomes disease [84,131].

Two observations qualify this framework. The interface is not confined to one animal, since maternal subclinical ketosis altered offspring metabolic preparedness and gut microbiome maturation [122]. And host mineral demand is itself community-shaping, hypophosphatemia selectively remodeling the hindgut [118], pairing with the hepatic response to prepartum phosphorus restriction [64]. At the finest resolution, the ketone body–butyrate–*HADHA* axis provides a single-cell endpoint for a microbially generated metabolite [55]. Together these interfaces justify modeling periparturient adaptation as one host–microbial system [36,38].

## 7. Multi-Omics Integration: From Associations to Causality

The evidence assembled above rests on qualitatively different measurement platforms whose outputs are not interchangeable. Amplicon (16S rRNA) sequencing profiles community composition but only weakly resolves function; shotgun metagenomics captures gene content and enables strain- and pathway-level inference but does not report activity; metatranscriptomics samples microbial expression; and untargeted or targeted metabolomics measures the chemical output that the host actually experiences. Discordances between compositional and functional readouts—for example, no compositional difference but clear metabolomic separation in hyperketonemia [107] or high Fusobacteriaceae abundance in clinically healthy uteri [127]—make clear that these platforms are complementary, not substitutable, and that function-first approaches (metagenome-assembled genomes, metatranscriptomics and paired metabolomics) are required to move from association to mechanism.

Causal inference likewise requires designs that go beyond correlation. Bayesian networks applied to paired host and microbial data [130], fecal microbiota transplantation into gnotobiotic mice [116,119], targeted single-taxon or metabolite supplementation [108] and single-nucleus resolution of host tissue [55] each contribute a different grade of evidence. Read together, they support treating periparturient adaptation as one causally coupled host–microbial system rather than two parallel descriptions.

## 8. Nutritional and Management Interventions

Three biomarker classes emerge. Host transcriptional markers accessible from blood include the IGF-1-associated leukocyte signature, the neutrophil recruitment deficit of metabolically stressed cows and the cross-tissue hubs *LBP*, *PTX3*, *S100A12* and *LCN2* [39,91,103]. Prepartum predictive markers are especially valuable because they permit intervention: adipose *CD209*, fecal community structure, teat apex *S. aureus* load and pre-clinical acylcarnitines [71,112,123,133]. Production and longevity markers include the eight hepatic hub genes explaining milk yield and the purine-metabolite mediators identified in long-lived cows [49,109]. Circulating miRNAs are a particularly attractive format, being stable, minimally invasive and already shown to discriminate metritis [90].

The interventions catalogued in Table 4 act at four levels. Prepartum energy density and body condition program hepatic and adipose capacity, restriction priming oxidative metabolism and overfeeding activating anabolic networks, with the optimum conditional on body condition [45,52,70]. Methyl donor and amino acid supply is mechanistically differentiated among choline, methionine and niacin and carries dose-dependent risk at the upper end [59,60,63], supported by evidence for rumen-protected limiting amino acids and choline [61] and for combined methionine and choline in lactational performance [62]. Micronutrient and mineral status acts through immune and structural routes alike: folate shows clear dose dependency [95,96] and is protective alongside selenium and vitamin E [24], while amino acid-complexed trace minerals outperform inorganic forms in leukocyte, endometrial and corium responses [86,100], consistent with integrated recommendations for vitamins, rumen-protected amino acids and trace minerals in mastitis prevention [87]. Fatty acid interventions are bidirectional, with conjugated linoleic acid reducing milk fat and hepatic lipid accumulation, oleic acid restraining lipolysis and linseed shifting mammary immune and metabolic pathways [74,79,139]. Direct microbial modulation completes the toolkit [132,136,137,138], while pegbovigrastim and appropriately timed vaccination round out the immunological space [92,93].

Several findings are actionable for breeding: fertility divergence, breed-specific immune strategy, hepatic hub genes predicting yield and accelerated hepatic aging all nominate selection targets [49,50,53,94]. Because microbial features are partly heritable and strongly associated with performance [35,109], microbiome-informed selection indices are a plausible medium-term extension.

**Table 4:** Nutritional and management interventions acting on the periparturient transcriptome and microbiome.

Intervention	Target Layer	Principal Molecular or Microbial Response	Production/Health Outcome	Reference
Selenium and vitamin E	Redox regulation	Improved antioxidant capacity across transition	Better periparturient health regulation	[23]
Selenium, vitamin E and folate	Antioxidant and immune status	Enhanced antioxidant defense and immune competence	Reduced incidence and severity of periparturient mastitis	[24]
Prepartum energy restriction vs. overfeeding	Liver transcriptome	*PPARA*, *SREBF1/2*, *SCAP*, *NFE2L2* activated by restriction; TAG synthesis by overfeeding	Restriction primes oxidative metabolism and stress resistance	[45]
Feeding level × body condition	Liver transcriptome	*BCAT1*, *GPX3*, taurine metabolism; *ALB*, *FABP7* under restriction in thin cows	Optimal-BCS cows’ benefit from restriction; thin cows from overfeeding	[52]
Rumen-protected choline vs. methionine	Liver transcriptome	1123 DEGs; *FGF21*, *SLC13A5*, *FBP2*, *CDH11*	Choline enhances metabolic efficiency; methionine modulates energy and immunity	[59]
Rumen-protected niacin vs. methionine	Liver transcriptome	1210 DEGs; *FADS2*, *SLC27A6*, *THRSP* up; *BCO2*, *S100A12* down	Niacin acts directly on lipid pathways; methionine via NAD/NADP routes	[60]
Rumen-protected limiting amino acids and choline	Systemic immunity and redox	Improved immune, antioxidative and anti-inflammatory status	Reduced periparturient inflammatory burden	[61]
Rumen-protected methionine + choline	Metabolism and lactation	Enhanced metabolic efficiency	Improved milk production performance	[62]
High-dose rumen-protected niacin	Liver transcriptome + miRNA	149 transcripts up (*HP*, *APCS*, *BOLA-DQA5*, *GPX4*); 338 down; 19 miRNAs down	Potential amplification of hepatic inflammation	[63]
Prepartum low-phosphorus diet	Liver transcriptome	*GNLY*, *CXCL3*, *SAA3*, *IL1A* prepartum; *SYT4*, *TRPC4*, *THRSP* postpartum	Safe strategy to mitigate hypocalcemia	[64]
Dry-period energy overfeeding	Adipose transcriptome	2434 prepartum DEGs; PPARγ, mTOR, *CXCR4*, *IGF1*	Anabolic priming; greater postpartum catabolic risk	[70]
Abomasal oleic acid	Adipose	PLIN5, PPARα, *SIRT3*, *SOD2* up; osteopontin down	Reduced lipolysis and improved insulin sensitivity	[74]
Linseed	Mammary + liver	Cell cycle and MHC class I up; lipid and carbohydrate metabolism down; hepatic *GLUT2* up	Higher milk lactose and altered fatty acid profile	[79]
Amino acid-complexed trace minerals	PMNL + endometrium	*DDX58*, *MPO*, *PLA2G4A*, *PPARG*; *TLR2/4*, *NFE2L2*, *SOD1*, *MUC1*	Faster involution and pathogen clearance	[86]
Vitamins, rumen-protected amino acids and trace minerals	Immune, antioxidant and anti-inflammatory axes	Coordinated enhancement of bovine immune responses	Prevention of periparturient mastitis	[87]
Vaccination timing	Whole blood	2235 DEGs before vs. 208 after calving; *ARG1* postpartum	Prepartum vaccination gives superior responsiveness	[92]
Pegbovigrastim	Leukocytes	*CD14*, *TLR2/4*, *MYD88*, *NLRP3*, *MMP9* up	Improved neutrophil adhesion and antimicrobial function	[93]
Coated folic acid (120 vs. 240 mg)	Lymphocyte transcriptome	TNF and NF-κB signaling; *CD4*, *CD8A* up at low dose; *SOCS3* down	Low dose gives balanced immune activation	[95]
Coated folic acid (low dose)	Lymphocyte metabolism	*DGAT2*, *ALOX5*, *GGH*, *ALDOA*, *TKT*; glycolysis and glutathione metabolism	Increased milk yield	[96]
Amino acid-complexed trace minerals	Corium	*BTD* up; *KRT5*, *CYBB* down; *PPARA*, *PPARD* up	Reduced heel horn erosion	[100]
Grape pomace	Uterine microbiome + mycobiome	*F. necrophorum*, *T. pyogenes*, *Candida* spp. reduced	Supported uterine microbial homeostasis	[132]
Naringin	Rumen resistome	Proteobacteria, ARGs and virulence factors reduced	Antimicrobial stewardship benefit	[136]
Phytosterols	Rumen microbiome	*Fibrobacter succinogenes*, GH13, glycolysis up	Greater microbial protein and propionate	[137]
*Brassica rapa* L./N-carbamoylglutamate	Rumen microbiome + metabolome	Cellulolytic taxa and SCFA up; arachidonic acid metabolism down	Higher milk yield and protein; less oxidative stress	[138]
Conjugated linoleic acid	Mammary, adipose and liver	Mammary lipid biosynthesis down; adipose and hepatic PPAR/AMPK/insulin pathways	Lower milk fat, NEFA and BHB; reduced type I ketosis risk	[139]

## 9. Nutritional Strategies That Modify the Microbiome and Host Transcriptome

A recurring theme across recent work is that several of the most effective interventions act through the pathway diet → ruminal (or hindgut) fermentation → microbial metabolite pool → hepatic and immune transcriptome. Phytosterol supplementation enriches Fibrobacter succinogenes and raises propionate supply, providing more glucogenic precursor to the liver during peak gluconeogenic demand [137]. Naringin and other phytochemicals contract the ruminal Proteobacteria fraction and lower antibiotic-resistance and virulence gene abundance, altering the LPS load presented to the host and, in turn, the hepatic acute-phase response [136]. Fiber-based strategies and alternative forages such as Brassica rapa L. expand cellulolytic taxa and raise short-chain fatty acids, which feedback on rumen-epithelial monocarboxylate transport and hepatic energy metabolism [138]. Grape pomace reshapes the uterine bacterial and fungal community, reducing *Fusobacterium necrophorum* and *Trueperella pyogenes* and thereby the inflammatory signal reaching the endometrial transcriptome [132]. One-carbon nutrients (methionine, choline and folate) and amino acid-complexed trace minerals act on the same axis from the host side by rewiring hepatic and leukocyte gene expression and modulating microbial substrate supply through altered digesta composition [59,60,61,62,86,95,96,140]. Reading these interventions through the nutrition → microbiome → transcriptome lens, rather than as a list, clarifies why divergent supplements can produce convergent phenotypic benefits and identifies where combined strategies are likely to be additive.

## 10. Biomarkers and Precision Dairy Farming

Three biomarker classes emerge, distinguished by sample source and intended application. Host transcriptional markers accessible from blood include the IGF-1-associated leukocyte signature, the neutrophil recruitment deficit seen in metabolically stressed cows and the cross-tissue hubs *LBP*, *PTX3*, *S100A12* and *LCN2* [39,90,102]. Prepartum predictive markers are especially valuable because they permit early intervention: adipose *CD209* expression, fecal community structure, teat apex *S. aureus* load and pre-clinical acylcarnitine profiles [70,111,122,132]. Production and longevity markers include the eight hepatic hub genes that explain variation in milk yield and the purine-metabolite mediators identified in long-lived cows [48,108]. Among these, circulating miRNAs stand out as a particularly attractive format, being stable, minimally invasive and already shown to discriminate metritis [89].

Several findings are actionable for breeding: fertility divergence, breed-specific immune strategy, hepatic hub genes predicting yield and accelerated hepatic aging all nominate selection targets [48,49,52,93]. Because microbial features are partly heritable and strongly associated with performance [35,108], microbiome-informed selection indices are a plausible medium-term extension.

Two conditions separate a candidate marker from a deployable one. The matrix must be sampleable at scale, which favors blood, milk, feces and teat-apex swabs over tissue biopsy and makes circulating microRNAs and leukocyte signatures the most tractable host readouts [89]. And the marker must retain its discrimination in commercial cohorts spanning several herds, diets and parities, at the scale used for uterine community profiling across twenty-four farms [123]. Neither condition is met by most of the markers listed above, which is the main obstacle to precision transition management rather than any shortage of candidates.

## 11. Limitations and Future Directions

Four constraints temper these conclusions. Sample size and biopsy burden: Many liver studies rest on five to seventeen animals per group [41,49,59,60], limiting power and inflating detected effect sizes, while minimally invasive alternatives [39,90] scale better but sacrifice tissue specificity. Bulk tissue averaging: Cell composition changes markedly around calving, so bulk DEGs may reflect shifting proportions rather than altered per-cell transcription, as laser-capture [85] and single-nucleus analyses [55] demonstrate. Taxonomy versus function: Much of the microbiome literature remains amplicon-based, and discordance between compositional and functional findings is conspicuous—no compositional difference but clear metabolomic separation in hyperketonemia [107], as well as high *Fusobacteriaceae* in healthy uteri [127] with no diversity difference yet distinct pathogen enrichment in clinical endometritis [125]—so function-first approaches such as metagenome-assembled genomes [108] and metatranscriptomics [137] should be prioritized. The strongest causal claims come from transfer and intervention experiments [55,108,116,117,119], which remain the exception, and murine models are imperfect proxies for a ruminant digestive system.

Addressing these constraints requires four priorities: first, standardized sampling frames, since comparability is compromised by windows spanning −49 to +28 days and consensus time points would enable the formal meta-analysis shown to be feasible by Dai et al. [103]; second, longitudinal paired designs sampling host tissue and microbial niches from the same animals at the same times, since almost all current crosstalk inference is assembled across studies; third, integration of the non-coding layer with microbial metabolites, given that miRNA and circRNA networks are nutritionally responsive [63,73] and sit where a metabolite signal would act; and Ffurth, translation to farm-deployable assays, since promising prepartum markers [71,112,123,133] require validation in commercial cohorts of the scale used by Basbas et al. [124]. Underlying all four is the need to treat genetic background [53,94], parity [50,84,131] and prepartum condition [48,52,110] as designed variables rather than nuisance covariates.

## 12. Conclusions

Periparturient adaptation is best understood as a coordinated, time-structured host transcriptional program executed against a simultaneously reconfiguring microbial background. On the host side, the evidence converges on a consistent architecture: hepatic prioritization of gluconeogenesis and lipid oxidation over storage, a self-limiting endoplasmic reticulum stress response in the first postpartum week, adipose transition from storage to lipolytic and inflammatory signaling, mammary redox mobilization through NFE2L2 and, finally, an immune program in which the capacity to resolve inflammation separates health from disease. On the microbial side, parturition and the dietary shift restructure ruminal, hindgut, uterine, vaginal and udder-surface communities in ways that alter glucogenic precursor supply, secondary bile acid production, glutathione precursor availability, barrier integrity and pathogen opportunity. The decisive advance of recent years is the demonstration that these layers are causally linked at named, testable nodes: a ruminal taxon supplying alanine to hepatic gluconeogenesis, microbial secondary bile acids setting monocyte competence, hindgut communities determining systemic glutathione status, ileal pathobionts driving hepatic steatosis and host tissue damage licensing uterine pathogen overgrowth. Reading transcriptomic and metagenomic evidence together therefore does more than add explanatory detail: it identifies intervention points that neither layer reveals alone and reframes transition management from the reactive detection of failed adaptation toward its prospective prevention.

## Figures and Tables

**Figure 1:**
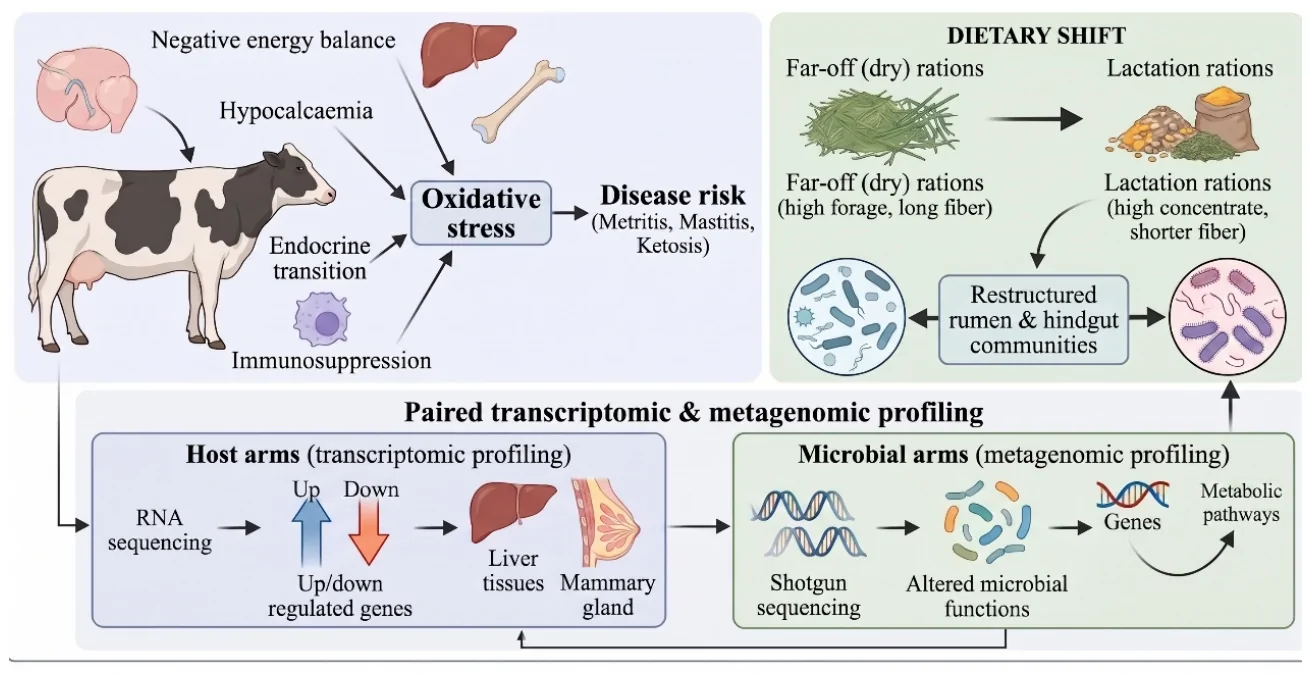
Physiological architecture of the periparturient period. Negative energy balance, hypocalcemia, endocrine transition and immunosuppression converge on oxidative stress and disease risk, while the shift from far-off to lactation rations restructures rumen and hindgut communities. Paired transcriptomic and metagenomic profiling captures both the host and microbial arms of this adaptation. Created in BioRender (https://BioRender.com/p9epsf0, accessed on 1 September 2026).

**Figure 2:**
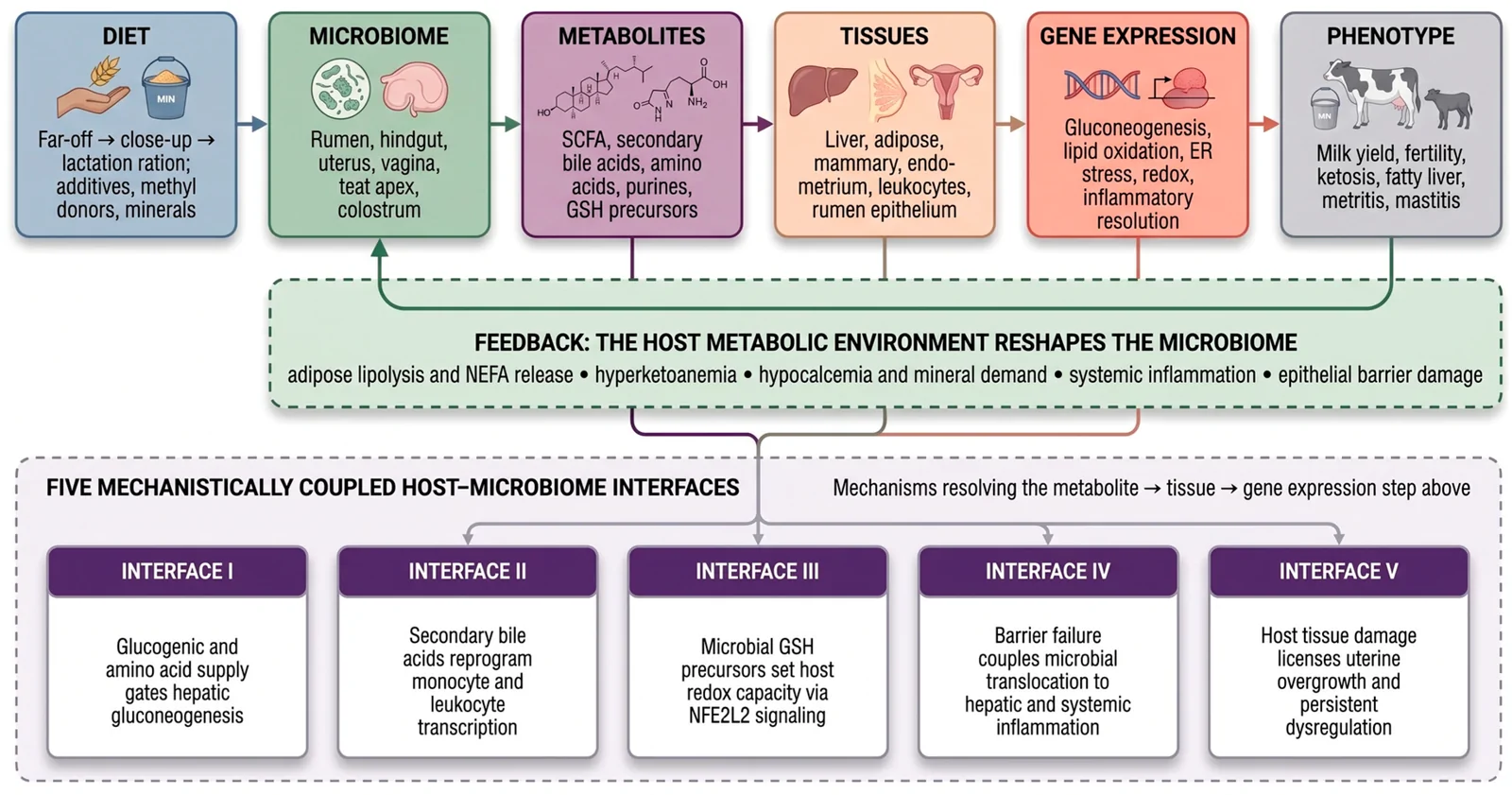
Integrated conceptual model of host–microbiome coupling during the transition period. The upper chain follows the sequence diet → microbiome → microbial metabolites → host tissues → gene expression → phenotype. Ration change and parturition restructure the rumen, hindgut, reproductive tract and udder-surface communities; fermentation, bile acid transformation and microbial amino acid synthesis determine the metabolite pool absorbed into portal and systemic blood; and that pool reprograms transcription in liver, adipose tissue, mammary gland, endometrium, leukocytes and rumen epithelium, with consequences for yield, fertility and disease. The green loop shows the return path, in which the host metabolic environment—adipose lipolysis and NEFA release, hyperketonemia, hypocalcemia and mineral demand, systemic inflammation and epithelial barrier damage—reshapes community composition and function.

**Table 1:** Literature search strategy, sources and selection criteria applied in this review.

Element	Specification
Review type	Narrative (integrative) review; no protocol registered, no PRISMA-based screening and no formal risk-of-bias assessment
Databases	PubMed/MEDLINE, Web of Science and Scopus; Google Scholar and ResearchGate used additionally to retrieve full texts and to identify sources not returned by the three indexed databases
Search date	Up to August 2026; no date restriction applied at the search stage
Population terms	Dairy cow, dairy cattle, periparturient, peripartum, transition period and early lactation
Platform terms	Transcriptome, RNA-seq, differential gene expression, microRNA, circRNA, metagenome, metagenomics, 16S rRNA, microbiota, microbiome and multi-omics
Context terms	Liver, adipose tissue, mammary gland, endometrium, leukocyte, rumen epithelium, hindgut, uterus, colostrum, negative energy balance, ketosis, fatty liver, metritis and mastitis
Inclusion criteria	English language; peer-reviewed journal indexed in the Science Citation Index Expanded; dairy cows sampled within a defined window relative to calving; host transcriptomic or host-associated microbial community data generated by sequencing; comparison or model and molecular outcome stated explicitly
Exclusion criteria	Conference abstracts, preprints and reports without full data; rodent, other ruminant and in vitro work, except where it establishes a mechanistic link with no bovine equivalent, in which case the model system is identified explicitly at that point
Hand searching	Reference lists of retrieved articles and of prior reviews screened manually
Prioritization	Anchored on 2020–2026 (93 of the 140 cited sources, 67%); earlier sources retained where required for foundational transition physiology and for pre-sequencing-era tissue transcriptomics
Synthesis	Interpretive and qualitative; no pooled effect estimates and no quantitative synthesis attempted

## Data Availability

No new data were created or analyzed in this study. Data sharing is not applicable to this article.
